# 2354: Pioneering the pathway with big data to eliminate hepatitis C viral infection (EHCV)

**DOI:** 10.1017/cts.2017.70

**Published:** 2018-05-10

**Authors:** Dawn A Fishbein, Ian Brooks, Emanuel Villa Baca, Ozgur Ozmen, Mallikarjun Shankar, Gil Weigand, Kristina Thiagarajan, Randy Estes, Alex Geboy, Hala Deeb, Mamta Jain, Lesley Miller

**Affiliations:** Georgetown - Howard Universities, Washington, DC, USA

## Abstract

OBJECTIVES/SPECIFIC AIMS: Hepatitis C viral (HCV) infections are rising significantly both in young adults and as newly diagnosed cases in “baby boomers.” New HCV therapeutics cure over 95% of cases, and a call has been made for elimination of the epidemic by 2030; yet major HCV cascade of care (CoC) barriers exist. We secured CTSA pilot funding to obtain preliminary data for an innovative clinical trial utilizing big data modeling toward HCV elimination. METHODS/STUDY POPULATION: Our pilot work has developed a coordinated, real-time clinical data management process across 3 major CTSA affiliated hospital systems (MedStar Health, Emory-Grady, and UT-Southwestern), and additional data will be obtained from a pragmatic clinical trial. Electronic medical records data will be mapped to the OHDSI model, securely transmitted to Oak Ridge National Laboratory, Knoxville, TN and exposed to integrated data, analytics, modeling and simulation (IDAMS). RESULTS/ANTICIPATED RESULTS: Our U01 CTSA application proposes that HCV-IDAMS will model modifications to the established HCV CoC at community and population levels and thus simulate future outcomes. As data volume increases, system knowledge will expand and recursive applications of IDAMS will increase the accuracy of our models. This will reveal real-world reactions contingent upon population dynamics and composition, geographies, and local applications of the HCV CoC. DISCUSSION/SIGNIFICANCE OF IMPACT: Only an innovative, integrated approach harnessing pragmatic clinical data, big data and supercomputing power can create a realistic model toward HCV elimination.

